# Dopamine D_1_ receptor stimulation modulates the formation and retrieval of novel object recognition memory: Role of the prelimbic cortex

**DOI:** 10.1016/j.euroneuro.2015.07.018

**Published:** 2015-11

**Authors:** Marie A. Pezze, Hayley J. Marshall, Kevin C.F. Fone, Helen J. Cassaday

**Affiliations:** a School of Psychology, University of Nottingham, Nottingham, UK; b School of Life Sciences, University of Nottingham, Nottingham, UK

**Keywords:** D_1_ receptors, SKF81297, Novel object recognition, Medial prefrontal cortex, Rat

## Abstract

Previous studies have shown that dopamine D_1_ receptor antagonists impair novel object recognition memory but the effects of dopamine D_1_ receptor stimulation remain to be determined. This study investigated the effects of the selective dopamine D_1_ receptor agonist SKF81297 on acquisition and retrieval in the novel object recognition task in male Wistar rats. SKF81297 (0.4 and 0.8 mg/kg s.c.) given 15 min before the sampling phase impaired novel object recognition evaluated 10 min or 24 h later. The same treatments also reduced novel object recognition memory tested 24 h after the sampling phase and when given 15 min before the choice session. These data indicate that D_1_ receptor stimulation modulates both the encoding and retrieval of object recognition memory. Microinfusion of SKF81297 (0.025 or 0.05 μg/side) into the prelimbic sub-region of the medial prefrontal cortex (mPFC) in this case 10 min before the sampling phase also impaired novel object recognition memory, suggesting that the mPFC is one important site mediating the effects of D_1_ receptor stimulation on visual recognition memory.

## 1 Introduction

Humans and other mammals have a natural tendency to explore novel stimuli such as those provided by novel environments or objects. For example, rats given the choice between a novel and familiar object spontaneously spend more time exploring the novel object. To discriminate between novel and familiar stimuli requires the ability to remember previously encountered stimuli (Ennaceur and Delacour, 1988). As this is an innate tendency, novel object recognition (NOR) procedures offer advantages over many learning and memory tasks in that NOR can be studied without training or the use of positive (e.g. food) or negative (e.g. foot shock) reinforcers (Lyon et al., 2012). Furthermore by selecting an appropriate phase for drug injection, the task can be utilised to distinguish effects on acquisition, consolidation and/or retrieval of visual memory (King et al., 2004).

The neurobiological substrates of recognition memory have been extensively studied (Winters et al., 2008, Warburton and Brown, 2010) and shown to require plasticity within the perirhinal cortex (Bussey et al., 1999, Brown and Aggleton, 2001). The prefrontal cortex (PFC) is also critically involved in object discrimination. While lesions to the medial prefrontal cortex (mPFC) particularly impair recognition memory requiring judgement about recency and object location (Hannesson et al., 2004), there is also an evidence that prefrontal mechanisms contribute to NOR based simply on object identity in rats. Recent fMRI studies show that disruption of mPFC activation is correlated with the extent of reduction in recognition memory (Zanto et al., 2011). Furthermore lesions of the mPFC in rats reduced memory for visual objects (Ragozzino et al., 2002), while anterior cingulate lesions in monkeys similarly impaired object memory processes including NOR (Meunier et al., 1997). In addition, electrophysiological recordings in the PFC have shown different patterns of neuronal activation dependent on the familiarity of the objects (Xiang and Brown, 2004).

It is also well established that dysfunction of dopamine (DA) neurotransmission disrupts recognition memory. For example, both acute and subchronic injection of the DA releasing agent, methamphetamine reduced NOR (Belcher et al., 2008, Camarasa et al., 2010). The mPFC is innervated by dopaminergic projections from the ventral tegmental area (VTA) (Lindvall et al., 1974) and we have previously shown that bilateral microinjection of the selective DA D_3_ receptor antagonist, S33084, into the PFC of rats caused a dose-related (0.025 or 0.05 μg/side) improvement, while injection of the preferential D_2_ receptor antagonist, L741,626, into the same region caused a dose-related (0.025 or 0.05 μg/side) impairment in NOR (Watson et al., 2012a, Watson et al., 2012b). While it is well established that DA D_1_ receptor modulation in the mPFC alters working memory (Goldman-Rakic, 1998), questions remain concerning its role in recognition memory. Besheer et al. (1999) reported that systemic administration of the D_1_ receptor antagonist, SCH23390 impaired NOR. Further studies suggest that D_1_ receptor activity in the mPFC is important for both the consolidation (Nagai et al., 2007, Rossato et al., 2013) and retrieval of long term recognition memory (Hotte et al., 2006). However, whether D_1_ receptor activation may also influence the acquisition of NOR has yet to be determined.

Therefore, the present study used systemic administration of the selective D_1_ receptor full agonist, R/S-(+/−)-6-chloro-7,8-dihydroxy-1-phenyl-2,3,4,5-tetrahydro-1H-3-benzazepine (SKF81297; Ki=2.2 nM; D_1_/D_2_ ratio >454.5, Nielsen et al., 1989), to explore the effect of D_1_ receptor stimulation on the encoding and expression of recognition memory after a short (10 min) and a long inter-trial interval delay (24 h). Second microinjection of SKF81297 into the prelimbic (PL) part of the mPFC prior to the acquisition of object memory was performed to determine whether short term recognition memory was dependent on mPFC DA D_1_ receptor activation.

## 2 Experimental procedures

### 2.1 Animals

Adult male Wistar rats (Charles River, UK) were caged in group of four on a 12:12 h light/dark cycle with food and water ad libitum. Rats were handled for approximately 10 min per day for 1 week prior to any procedure. All procedures were carried out in accordance with the United Kingdom (UK) Animals Scientific Procedures Act 1986, Project Licence number: PPL 40/3163. In experiments 1 (*n*=36), 2 (*n*=35), 3 and 5 (*n*=36), non-naive rats (mean weight 431 g) were used, in each case counterbalanced for previous experimental experience. In experiments 4 and 6, 11 naive rats (mean weight 297 g; operated in the range 270–340 g) were used. One rat was excluded from the analysis because it escaped from the NOR box during both the sampling and choice phase.

### 2.2 Systemic injection procedure

In experiments 1 and 2, saline or the selective D_1_ agonist SKF81297 (0.4 or 0.8 mg/kg s.c.) was injected 15 min before the sampling phase of the NOR procedure (see below). In experiments 3 and 5, the same doses were administered 15 min before the NOR testing phase and immediately prior to exposure to the locomotor activity boxes respectively. Drug doses were those used in a previous study in our laboratory (Nelson et al., 2012). SKF81297 hydrobromide (Tocris, UK) was dissolved in saline (0.154 M sterile NaCl) on each test day with the pH adjusted to 7 using 0.1 M NaOH.

### 2.3 Implantation of guide cannulae into the mPFC

Rats (*n*=11) were implanted with bilateral infusion guide cannula consisting of a 5 mm plastic pedestal holding two 26 gauge metal tubes 1.2 mm apart and projecting 4.5 mm from the pedestal was implanted through small holes drilled in the skull. The tips of the guide cannula were aimed 0.5 mm above the injection site in the PL part of the prefrontal cortex, at the following coordinates (Paxinos and Watson, 1998): 3 mm anterior, ±0.6 mm lateral from bregma and 3.8 mm ventral from the skull surface. For additional information see Supplementary material.

### 2.4 Microinjection procedure

Rats were gently restrained and 33 gauge injectors (Plastic Ones, Bilaney, UK) inserted into the guides such that tips extended 0.5 mm into the mPFC, and the injector ends were connected through polyethylene tubing to 5-µl syringes mounted on a microinfusion pump (model SP200iZ, World Precision Instruments). A volume of 0.5 μl/side of 0.154 M saline or SKF81297 (0.025 or 0.05 μg/side) was then infused bilaterally over 1 min. The movement of an air bubble, which was included in the tubing, was monitored to verify that liquid was successfully infused into the brain. The injector remained in place for one additional minute to allow for tissue absorption of the infusion bolus. The injectors were then removed and the stylets replaced. The choice phase of novel object discrimination (NOD) commenced 10 min after infusion. Locomotor testing began as soon as possible after the infusion and the time course of activity was followed for 60–90 min to determine the optimal onset of the drug action. In experiments 4 and 5b, SKF81297 used was dissolved in saline at a concentration of 0.05 μg/0.5 μl. This solution was aliquoted and kept frozen until use. On the day of infusion an aliquot was thawed and a part of this solution was diluted to a concentration of 0.025 μg/0.5 μl with saline.

### 2.5 NOR: behavioural apparatus

All testing was conducted in a 38×40 cm opaque plastic rectangular arena with 54 cm high walls. The stimuli consisted of duplicate copies of bottles and flasks made of glass, metal or plastic of varied shape, colour and size which were too heavy to be displaced by the animal (Ennaceur and Delacour, 1988), for more information also see Supplementary material. The rats׳ behaviour was recorded and later analysed.

### 2.6 NOR: behavioural procedure

The NOR task was based on previously established protocols (Nelson et al., 2010, 2011). One day before the test day, animals received an acclimatisation session. The rats were placed individually into the arena for 1 h. On the following day, rats underwent a re-acclimatisation of 3 min to the arena. In experiments 1, 2 and 3, different groups of rats were injected with saline, 0.4 or 0.8 mg/kg of SKF81297 (s.c.) and returned to their home cage for 15 min. The rats were then given one 5 min sample phase in which the animals were allowed to explore two identical copies of the sample object. In experiment 3, rats were exposed to 5 min sample phase directly after the re-acclimatisation. The total time spent exploring the two identical objects was recorded. After a delay of 10 min (experiment 1) or 24 h (experiments 2 and 3), in which rats were return to their home cage, each rat was replaced for 3 min in the arena, which now contained a novel object and an identical copy of the object previously seen during the sampling phase. In every experiment, at the sampling phase the object exploration time was scored as the total over the full 5 min exposure to the objects. For the choice sessions, the time spent exploring the familiar and novel object was recorded over a total of 3 min. Because the preference for novel object, objects can decline rapidly, these exploration times were scored in three 1-min blocks.

Comparison between the systemic drug studies (experiments 1–3) provides the temporal resolution to distinguish effects at encoding versus retrieval in the NOR procedure. Since both encoding and retrieval were affected, the experiment 4 infusion study used the experiment 1 timeline (infusion before the sample phase and a 10 min retention interval) which does not distinguish effects on encoding and retrieval. Experiment 4 was run over a 6 day cycle in a within-subjects design to reduce the number of animals subjected to the cannulation procedure. On days 1, 3 and 5 rats were placed individually into the arena for 1 h. On days 2, 4 and 6, rats underwent a re-acclimatisation of 3 min to the arena before receiving one of three bilateral mPFC infusions 10 min before the sampling phase: saline, 0.05 μg (0.025 μg/side), 0.1 μg (0.05 μg/side) of the selective D_1_ receptor agonist SKF81297. The order of the three infusions was counterbalanced using a Latin square design. During the 5 min sampling period the rats were exposed to two identical objects. After a delay of 10 min in which the rats were returned to their home cage, each rat was tested for 3 min in the arena containing a novel object and an identical copy of the object previously seen during the sampling phase. Again, the time spent exploring the familiar and novel object was recorded. In the course of the three sampling/choice sessions, three different object pairs were used, counterbalanced across the infusions conditions.

#### 2.6.1 Inter-rater reliability

An independent experimenter who was blind to the treatment and object contingencies rescored 20% of all test phases from the original video recording. The rescored results significantly correlated with the original scores (*r*=0.85, *p*<0.0001) indicating robust inter-rater reliability.

### 2.7 Locomotor activity: behavioural apparatus

Locomotor activity was assessed as described by Pezze et al. (2014), and in the Supplementary material.

### 2.8 Locomotor activity: experimental designs

The locomotor effects of SKF81297 were tested in between-subjects designs. One day before the injection or infusion each rat was placed in a test chamber for 1 h, to obtain baseline measures of activity, and also to habituate the animal to the box. These baseline measures of activity were used to match activity levels across the injection or infusion group allocations for the subsequent tests of the locomotor effects of SKF81297. On the following day, rats were replaced in the same test chamber for 30 min to achieve further habituation and so maximise the ability to detect any SKF81297-induced locomotor hyperactivity. Rats were then subcutaneously injected with saline, 0.4 or 0.8 mg/kg of SKF81297 in experiment 5, or microinfused with saline, 0.025 or 0.05 μg/side into the mPFC in experiment 6, and immediately replaced in the activity box for 60 min.

### 2.9 Verification of cannulae placements for behavioural studies

Histology was assessed as described by Pezze et al. (2014), and in the Supplementary material.

### 2.10 Design and analysis

The results are shown as means (±SEM); *p* Values of less than 0.05 are considered to indicate statistical significance.

#### 2.10.1 Exploration time

In experiments 1–3, choice exploration times were analysed in a mixed design using treatment as the between-subjects factors. Object (novel versus familiar) was a within-subjects factor as was 1-min block (at 3 levels). Thus, this design examines minute-by-minute variation in NOR over the duration of the test session. The results obtained using the Latin Square procedure of experiment 4 were analysed in an entirely within-subjects design, in the same way as experiments 1–3 but in this case treatment was also within-subjects. ANOVAs showed marked variation in NOR over the 3 min of test (as reflected in significant interactions between object and 1-min block). Therefore, based on the results obtained in experiments 1 which consistently indicated that the preferential object exploration was greater in the first min of the choice trial, follow up analyses were restricted to the first min. Where ANOVA showed significant interactions, separate follow-up analyses of the familiar or novel object exploration time were performed using treatment as the factor. Post-hoc pairwise comparisons were performed using Fisher׳s PLSD. On the basis of the pattern of results of the first experiment, planned comparisons requiring a paired comparisons *t*-tests were systematically used to test for NOD.

#### 2.10.2 Discrimination ratio

The time spent exploring the novel object divided by the total time spent exploring both objects was calculated for the first min and total three min of exploration. In experiment 1–3 the data were subjected to ANOVA with drug treatment as a between-subjects factor. In experiment 4 drug treatments was a within-subject factor. Effects revealed by the ANOVA were further analysed using Fisher׳s PLSD test. One sample *t*-tests were used to compare performance measures to the 0.5 value of the ratio which reflects chance level performance.

#### 2.10.3 Locomotor activity

In experiments 5 and 6 the activity levels were examined in a mixed design with doses as the between-subjects factor and 10 min blocks (locomotor activity) as the within-subjects factor.

## 3 Results

### 3.1 Experiment 1: spontaneous NOR after a delay of 10 min – systemic injection of SKF81297 before the sampling phase

ANOVA showed that SKF81297 attenuated the total exploration of both objects during both the sample (*F*(2,33)=15.81; *p*<0.0001) and choice phases (*F*(2,33)=6.17; *p*<0.006) of the NOD test when given by systemic administration (Table 1). Post-hoc analysis showed that both doses of SKF81297, 0.4 and 0.8 mg/kg, significantly reduced the total object exploration time during the sample phase (*p*<0.01 and *p*<0.001 from saline, respectively) and the highest dose reduced exploration during the choice phase (*p*<0.05) (Table 1).

**Figure 1:**
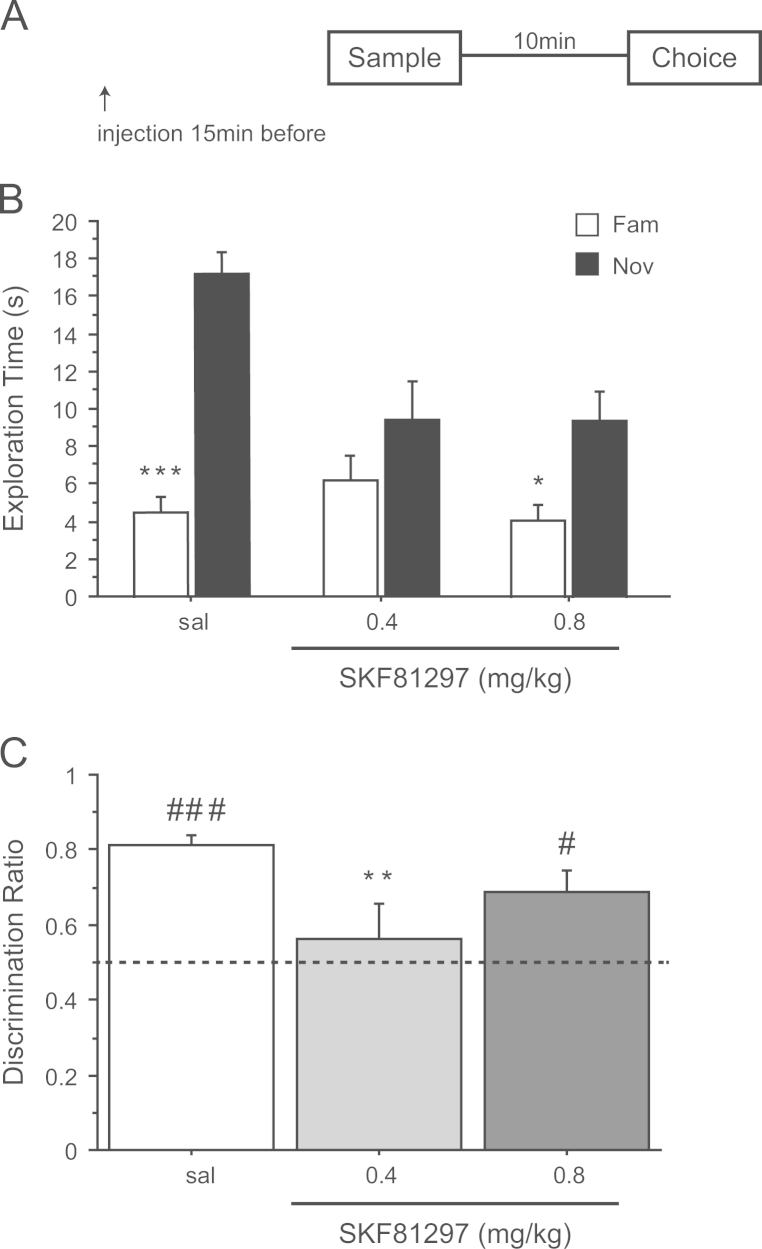
Effect of saline (sal) or SKF81297 (0.4 and 0.8 mg/kg) injected before the sample phase on novel object recognition after a retention delay of 10 min. (A) Timeline of experiment 1 to illustrate the 10 min retention delay between the sample and the choice phase and the timing of the injections 15 min before the sample phase. (B) Effect of SKF81297 on the first min of exploration of a novel (Nov) and a familiar (Fam) object during the choice phase. *p<0.05, ***p<0.0001, significant difference as compared to familiar object. (C) Choice performance presented as discrimination ratio; stippled line indicates chance. **p<0.001, significantly different from the saline group; ^#^*p*<0.05, ^###^*p*<0.0001 significantly different from chance. Data are shown as a mean±S.E.M (*n*=12 rats per group).

A minute by minute change in object exploration over the 3 min test period of the choice trial was revealed by a three-way interaction of treatment×object×1-min block (*F*(4,66)=2.89; *p*<0.029). Inspection of the means indicated that the effect of SKF81297 was due to a decrease of the time spent exploring the novel object during the first minute of the choice trial (Fig. 1A). ANOVA of exploration time during the first 1-min block yielded a significant interaction of object×treatment (*F*(2,33)=8.49; *p*<0.002). This interaction was however not significant during the second (*F*(2,33)=0.79; *p*=0.46) and the third (*F*(2,33)=1.34; *p*=0.27) 1-min blocks.

Follow-up analyses of the time spent exploring the novel object during the first 1-min block also showed a main effect of drug (*F*(2,33)=7.55; *p*<0.002). This arose because of decreased novel object exploration compared to saline after both low (sal vs. 0.4 mg/kg *p*<0.05) and high doses (sal vs. 0.8 mg/kg *p*<0.05) of SKF81297. There was no significant effect of drug on familiar object exploration (*F*(2,33)=1.32; *p*=0.28). Furthermore, planned comparison (by paired *t*-test) showed that the exploration times during the first 1-min block were significantly higher for the novel object than for the familiar object in the control group (*t*(11)=15.12; *p*<0.0001) and after an injection of 0.8 mg/kg of SKF81297 (*t*(11)=2.85; *p*<0.05), but not after injection of 0.4 mg/kg (*t*(11)=1.52; *p*=0.15) (Fig. 1B).

It was also clear from the discrimination ratios that during the first 1-min block the drug groups differed markedly in the proportion of time spent exploring the two objects (Fig. 1B). ANOVA of the 1 min discrimination ratio confirmed a significant main effect of SKF81297 (*F*(2,32)=3.82; *p*<0.04). Post-hoc comparisons indicated that the lower (*p*<0.05), but not the higher dose (*p*=0.16) of SKF81297, decreased recognition of the novel object compared to saline. This was also confirmed by one sample *t-*tests comparing the discrimination ratio in each group to chance (0.5). Performance differed from chance in the saline group (*t*(11)=11.29; *p*<0.0001) and the group receiving 0.8 mg/kg (*t*(11)=3.23; *p*<0.05), but not in the group receiving an injection of 0.4 mg/kg of SKF81297 (*t*(10)=0.70; *p*=0.49).

These data indicate that systemic activation of D_1_ receptor before encoding impaired the ability of the rats to discriminate novel from familiar object using a trial delay of 10 min.

### 3.2 Experiment 2: spontaneous NOR after a delay of 24 h – systemic injection of SKF81297 before the sampling phase

In this experiment, SKF81297 affected exploration time during the sample (*F*(2,13)=5.10; *p*<0.03) but not the choice (*F*(2,13)=0.32; *p*=0.73) phase after systemic administration before object encoding (Table 1). Post-hoc analysis showed that both doses of SKF81297 significantly reduced the total object exploration time during the sample phase (sal vs. 0.4 mg/kg *p*<0.05 and sal vs 0.8 mg/kg *p*<0.05).

**Figure 2:**
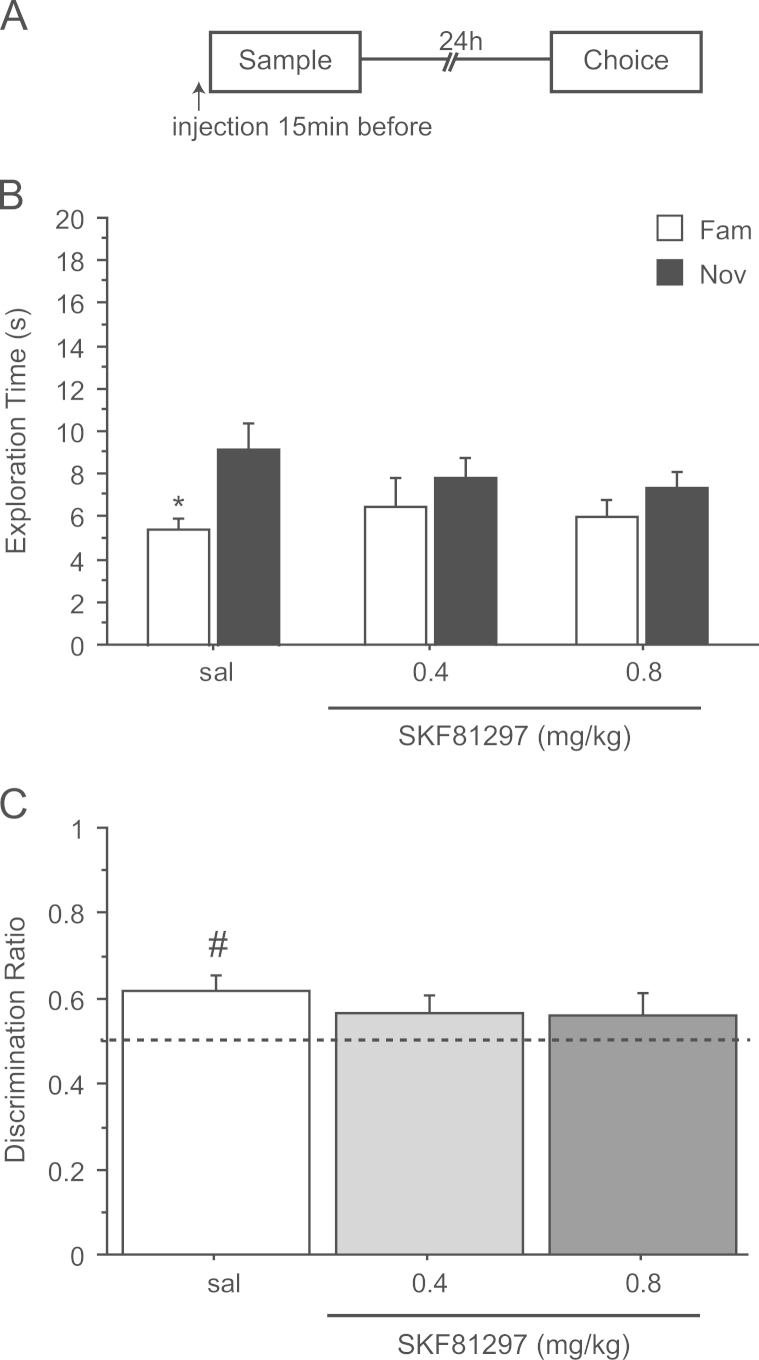
Effect of saline (sal) or SKF81297 (0.4 and 0.8 mg/kg) injected before the sample phase on novel object recognition after a retention delay of 24 h. (A) Timeline of experiment 2 to illustrate the 24 h retention delay between the sample and the choice phase and the timing of injections 15 min before the sample phase. (B) Effect of SKF81297 on the first min of exploration of a novel (Nov) and a familiar (Fam) object during the choice phase.*p<0.05, significant difference compared to familiar object. (C) Choice performance presented as discrimination ratio; stippled line indicates chance. ^#^*p*<0.05, significantly different from chance. Data are shown as a mean±S.E.M (*n*=11-12 rats per group).

There was no interaction between object, drug and 1-min block (*F*(4,64)=1.53; *p*=0.20). However, there was a main effect of object (*F*(1,32)=9.80; *p*<0.004) reflecting a preferential exploration of the novel object and a main effect of 1-min block (*F*(2,64)=47.52; *p*<0.0002), consistent with greater exploration of the novel object during the first min (*F*(1,32)=8.32; *p*<0.007) but not during the second (*F*(1,32)=3.57; *p*=0.06) and third minutes (*F*(1,32)=0.07; *p*=0.79). Accordingly, and for comparison with experiment 1, planned comparisons of the novel versus familiar object were restricted to the first minute only. These showed that rats in the saline group explored the novel object (*t*(11)=2.79; *p*<0.05) more than rats injected with 0.4 mg/kg (*t*(10)=1.06; *p*=0.31) and 0.8 mg/kg (*t*(11)=1.08; *p*=0.30) SKF81297 confirming that the main effect of SKF81297 was restricted to early exploration (Fig. 2A).

The discrimination ratio during the first 1-min of testing showed SKF81297 treated rats explored both objects equally, whereas the saline group tended to show a preference for the novel object (Fig. 2B). Even though there was no significant effect of drugs on the discrimination ratio during the 1-min block (*F*(2,32)=0.57, *p*=0.56) only the performance of the control group was better than chance (*t*(11)=3.23; *p*<0.05).

These data indicate that systemic activation of the D_1_ receptor transmission before the encoding tended to impair the recognition of a novel object after a delay of 24 h.

### 3.3 Experiment 3: spontaneous NOR after a delay of 24 h – systemic injection of SKF81297 before the choice phase

The groups were well matched for exploration time during the sample phase (*F*(2,33)=0.815; *p*=0.45) but exploration was decreased under SKF81297 during the choice phase (*F*(2,33)=19.19; *p*<0.0001) (Table 1). Post-hoc analysis showed that both doses of SKF81297 significantly reduced the total object exploration time during the 3 min choice phase (sal vs. 0.4 mg/kg *p*<0.05 and sal vs. 0.8 mg/kg *p*<0.05).

**Figure 3:**
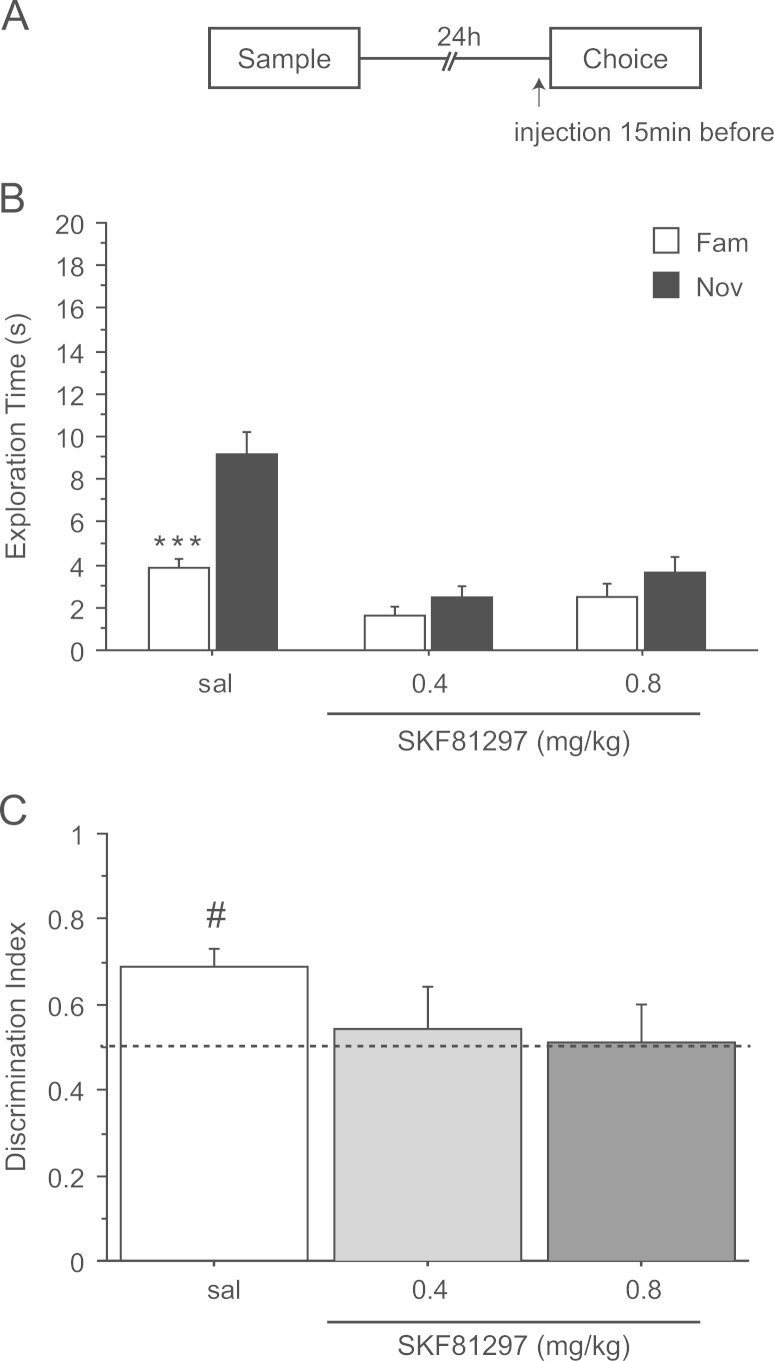
Effect of saline (sal) or SKF81297 (0.4 and 0.8 mg/kg) injected before the choice phase on novel object recognition after a retention delay of 24 h. (A) Timeline of experiment 3 to illustrate the 24 h retention delay between the sample and the choice phase and the timing of injections 15 min before the choice phase. (B) Effect of SKF81297 on the first min of exploration of a novel (Nov) and a familiar (Fam) object during the choice phase. ***p<0.0001, significant difference compared to familiar object. (C) Choice performance presented as discrimination ratio; stippled line indicates chance. ^#^p<0.05, significantly different from chance. Data are shown as a mean±S.E.M (*n*=12 rats per group).

A significant interaction between treatment×object×1-min block (*F*(4,66)=3.38; *p*<0.02) suggests that the effects on object exploration changed over the 3 min of the choice trial. Examination of the means confirmed that NOR was again more marked during the first minute of exploration (Fig. 3A) and prompted further analysis by each 1-min block. ANOVA of exploration time during the first 1-min block yielded a significant interaction of object×treatment (*F*(2,33)=7.40; *p*<0.003), suggesting that the drug preferentially reduced novel object exploration time. This interaction was not significant during the second (*F*(2,33)=0.84; *p*=0.44) and the third (*F*(2,33)=0.47; *p*=0.63) 1-min blocks.

Follow-up analysis of the time spent exploring the novel object during this first 1-min block showed that both 0.4 (sal vs. 0.4 mg/kg *p*<0.0001) and 0.8 mg/kg (sal vs. 0.8 mg/kg *p*<0.0001) of SKF81297 decreased novel object exploration time. While there was also a significant effect of drug on familiar object exploration (*F*(2,33)=5.04; *p*<0.02), this effect only reached significance after injection of 0.4 mg/kg SKF81297 (sal vs. 0.4 mg/kg *p*<0.05; sal vs. 0.8 mg/kg *p*=0.07). Planned comparisons demonstrated that the exploration times were significantly higher for the novel than the familiar object in the control group (*t*(11)=3.93; *p*<0.001), but not after 0.4 mg/kg (*t*(11)=1.63; *p*=0.13) or 0.8 mg/kg of SKF81297 (*t*(11)=1.51; *p*=0.15) (Fig. 3A).

Both doses of SKF81297 tended to reduce the discrimination ratio compared to saline during the first 1-min of the choice trial (Fig. 3B) but there was no significant effect of drug (*F*(2,32)=1.43, *p*=0.25), However, planned comparison showed that performance in the saline-injected control group was greater than chance (*t*(11)=4.23; *p*<0.05), whereas it was not with either 0.4 mg/kg (*t*(10)=0.4; *p*=0.69) or 0.8 mg/kg (*t*(11)=0.11; *p*=0.91) of SKF81297. Thus these data suggest that the retrieval of NOR is also disrupted by DA D_1_ receptor stimulation.

### 3.4 Experiment 4: spontaneous NOR after a delay of 10 min – mPFC infusion of SKF81297 before the sampling phase

ANOVAs of the time spent exploring both objects did not reveal any differences between groups during the sample phase (*F*(2,18)=1.42; *p*=0.26) but showed a reduction in total exploration during the choice phase (*F*(2,18)=4.86, *p*<0.03) with injection of SKF81297 into the mPFC (Table 1). Post-hoc analysis revealed that only the lower dose reduced exploration during the choice phase (sal vs. 0.025 μg/side *p*<0.05 and sal vs. 0.05 μg/side *p*=0.16; Table 1).

**Figure 4:**
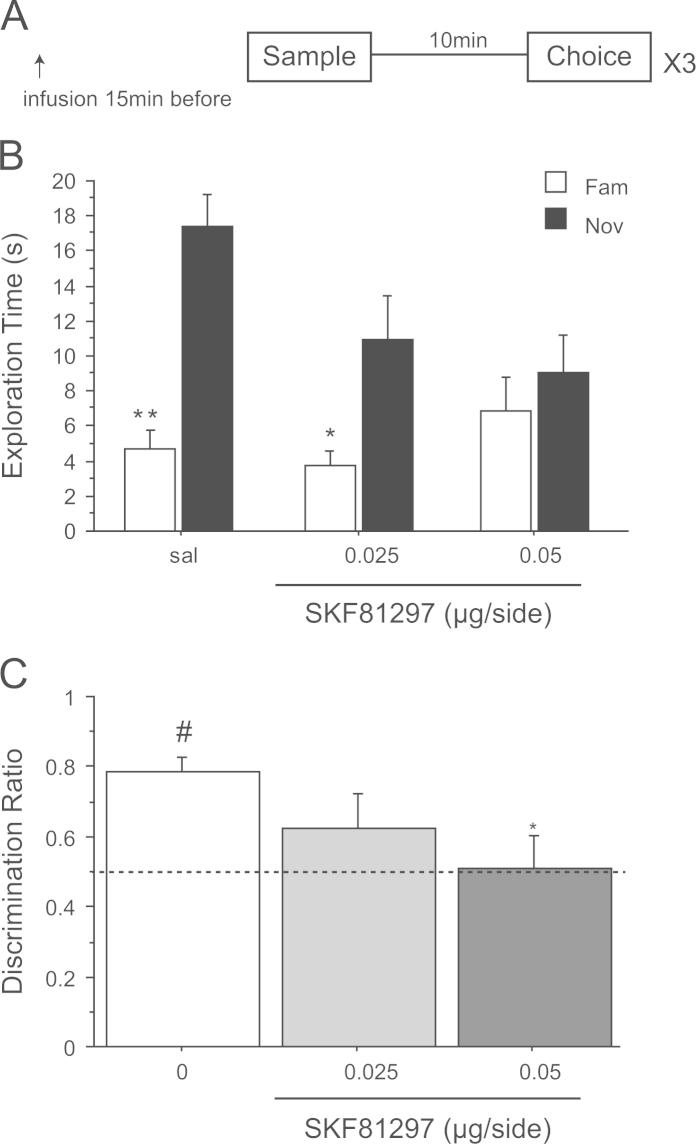
Effect of saline (sal) or SKF81297 (0.025 and 0.05 μg/side) infused into the prelimbic cortex before the sample phase on novel object recognition after a retention delay of 10 min. (A) Timeline of experiment 4 to illustrate the 10 min retention delay between the sample and the choice phase, the timing of the infusions 15 min before the choice phase and the repeated (×3) testing in the within-subjects design. (B) Effect of SKF81297 on the first min of exploration of a novel (Nov) and a familiar (Fam) object during the choice phase. *p<0.05, **p<0.001, significant difference as compared to the familiar object. (C) Choice performance presented as discrimination ratio; stippled line indicates chance. *p<0.01, significantly different from the saline group; ^#^*p*<0.05, significantly different from chance. Data are shown as a mean±S.E.M (*n*=10 rats in total).

There was a 3 way interaction of treatment×object×1-min block in the linear trend (*F*(1,9)=7.37; *p*<0.03). Fig. 4B shows that intra-mPFC infusion of SKF81297 decreased novel object preference during the first minute of the choice phase. Accordingly follow-up analyses were restricted to the first 1 min where ANOVA showed a very strong trend of object×treatment (*F*(2,18)=3.50; *p*=0.05) while novel object preference in the 2-min (*F*(2,18)=1.62; *p*=0.22) and 3-min blocks (*F*(2,18)=0.49; *p*=0.95) was unaffected. Analysis of the 1 min novel and familiar object exploration times separately showed a main effect of drug (*F*(2,18)=4.64; *p*<0.03). Furthermore novel object exploration time was decreased compared to the saline group after mPFC-infusion of SKF81297 at 0.025 μg/side (*p*<0.05) and 0.05 μg/side (*p*<0.05). On the contrary, the exploration time of the familiar object was unaffected by this treatment (*F*(2,18)=1.62; *p*=0.22). Planned comparisons demonstrated that the exploration times between the novel and the familiar object were significantly different in the saline group (*t*(9)=5.94; *p*<0.001) and after an infusion of 0.025 μg/side of SKF81297 (*t*(9)=2.40; *p*<0.05). However at 0.05 μg/side, there was no difference (*t*(9)=0.80; *p*=0.44) (Fig. 4A).

The discrimination ratio analysis for the first 1-min block also yielded a significant main effect of dose (*F*(1,9)=8.87; *p*<0.002). Post-hoc comparisons revealed a significant decrease in performance after infusion of 0.05 μg/side of SKF81297 compared to the control group (sal vs. 0.025 μg/side *p*<0.05; sal vs. 0.05 μg/side *p*=0.16) (Fig. 4C). Additional evidence from one sample *t*-tests showed that only the performance of the saline group was different from chance (chance vs. saline *t*(9)=6.92; *p*<0.0001; chance vs. 0.025 μg/side *t*(9)=1.29; *p*=0.23; chance vs. 0.05 μg/side *t*(9)=0.18; *p*=0.86) (Fig. 4B). Together these data suggest that activation of DA D_1_ receptors in the mPFC during encoding impairs object discrimination using a delay of 10 min.

### 3.5 Experiment 5: systemic injections of SKF81297 and locomotor activity

**Figure 5:**
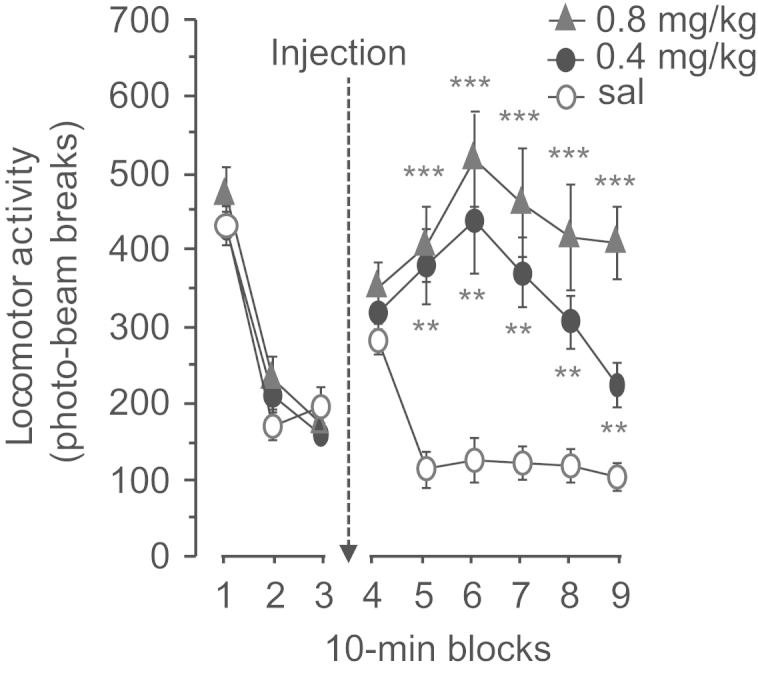
Effect of systemic injections of SKF81297 on spontaneous activity. The rats were habituated to the activity chambers for 30 min before being injected with saline, SKF81297 0.4 or 0.8 mg/kg of SKF81297. Locomotor activity was then monitored for an additional 60 min. Data are presented as mean±S.E.M. (*n*=12 rats per group). Asterisks indicate a significant difference as compared to saline with **p<0.001, ***p<0.0001.

SKF81297 markedly increased locomotor activity in the activity boxes (Fig. 5). In the 30 min preceding the injection of saline or SKF81297 (0.4 and 0.8 mg/kg), the different infusion groups showed similar locomotor activity. There was no main effect of treatment: (*F*(2,33)=0.55; *p*=0.56) nor any interaction of treatment×10 min time bin (*F*(4,66)=2.22; *p*=0.07). Systemic administration of SKF81297 increased activity at all doses compared to saline starting 10 min after injection and lasting for about 50 min. There was both a main effect of treatment (*F*(2,33)=15.9; *p*<0.0001) and an interaction of treatment×10 min bin (*F*(10,165)=6.41; *p*<0.0001). Separate ANOVAs and post-hoc comparisons of locomotor activity during the 6 10-min blocks revealed that, compared to saline, 0.4 and 0.8 mg/kg SKF81297 did not increase locomotor activity during the first 10-min bin (*F*(2,33)=1.68; *p*=0.20) whereas groups differed during the other 10-min bins (all *F*>10.85; *p*<0.002; post-hoc tests, *p*<0.05).

### 3.6 Experiment 6: infusion of SKF81297 into the mPFC and locomotor activity

**Figure 6:**
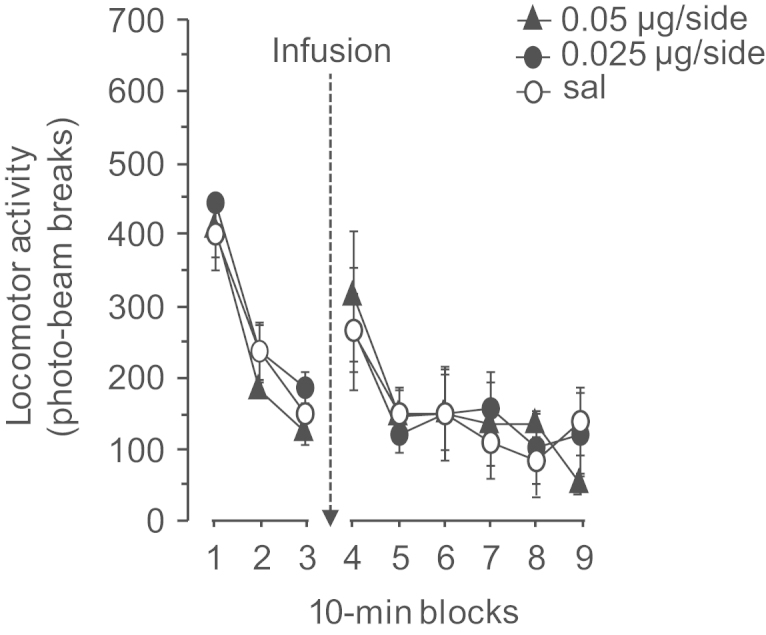
Effect of PL infusion of SKF81297 on spontaneous activity. The rats were habituated to the activity chambers for 30 min before being injected with saline (*n*=4), 0.025 (*n*=3) or 0.05 μg/side (*n*=4) of SKF81297. Locomotor activity was then monitored for an additional 60 min. Data are presented as mean±S.E.M.

SKF81297 infusion into the mPFC did not affect locomotor activity in the activity boxes (Fig. 6). The different infusion groups showed similar locomotor activity in the 30 min preceding the infusion of saline or SKF81297 (0.025 or 0.05 μg/side). There was no main effect of treatment (*F*(2,8)<1) nor any interaction of treatment×10 min bin: (*F*(4,16)<1). Following infusion there was still no main effect of treatment (*F*(2,8)<1) or interaction of treatment×10 min bin (*F*(10,40)<1).

### 3.7 Histology

**Figure 7:**
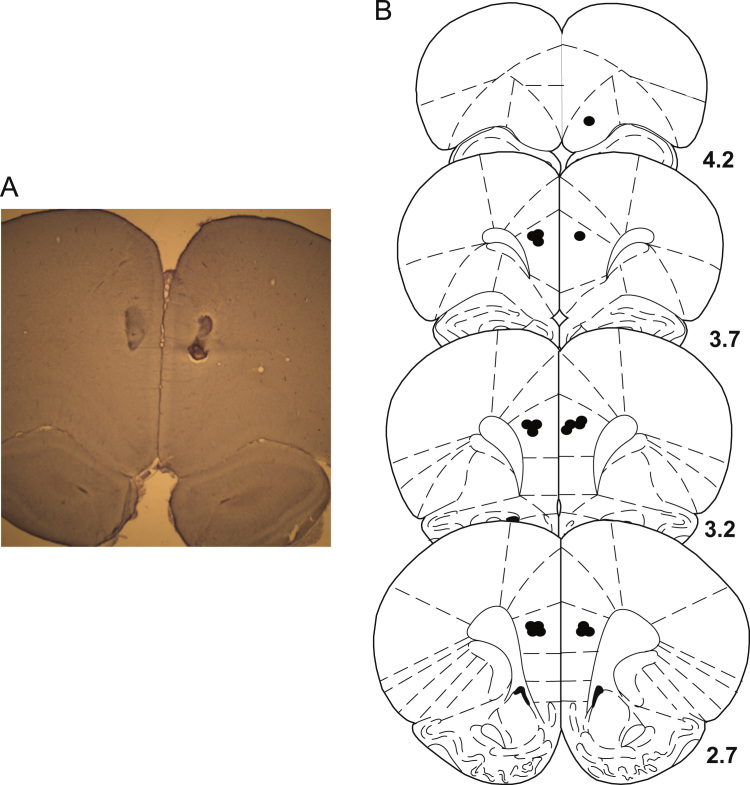
Infusion cannulae placement. (A) Cresyl-violet-stained section showing an example of PL infusion site. (B) Approximate locations of infusion cannula tips (dots) in the PL subdivision of the mPFC. Locations are shown on coronal plates adapted from Paxinos and Watson (1998), with numbers indicating distance anterior from bregma in millimetres.

No gross damage was seen after drug infusion, and the morphological structure of the mPFC was preserved (Fig. 7A). Fig. 7B shows reconstructed injector tip placements which were between 2.7 and 4 mm anterior to bregma in in the PL region of the mPFC according to the atlas of Paxinos and Watson (1998).

## 4 Discussion

Systemic (s.c.) administration of the D_1_ receptor agonist SKF81297 before encoding impaired NOR at the lowest dose after a delay of 10 min. Both doses of SKF81297 (s.c.) decreased NOR encoding and retrieval after a delay of 24 h. Both doses of SKF81297 (s.c.) stimulated locomotor activity. In experiment 4, we used a 10 min delay to show that intra-mPFC infusion of SKF81297 induced a dose-related impairment in encoding and/or retrieval. Both overall object exploration and locomotor activity were unaffected by this manipulation. Thus the present study shows that DA D_1_ receptor activation modulates the formation and expression or retrieval of visual recognition memory and that this effect is, at least in part, mediated in mPFC. In contrast, the effects of DA D_1_ receptor activation on general levels of activity and exploration are not necessarily mediated in mPFC.

### 4.1 Effect of DA D_1_ receptor stimulation on locomotor activity and exploration

Increased locomotor activity after s.c. administration of the D_1_ receptor agonist SKF81297 is consistent with previous reports (Arnt et al., 1992). Similarly,intra-mPFC infusion of SKF81297 has previously been reported to be without effect on locomotor activity (Sorg et al., 2004). Independent evidence suggests that the increased locomotor activity observed after s.c. administration of SKF81297 may have been induced by its stimulating effect on accumbal and/or striatal D_1_ receptor-mediated transmission (Cools et al., 2002, Diaz Heijtz and Castellanos, 2006).

General locomotor activity levels do not necessarily predict object exploration. As can be seen from comparison of the saline groups, there were different baseline levels of exploration, but each experiment controls for these unexplained shifts (sometimes particular batches of animals are more or less exploratory for reasons which are poorly understood, and with respect to the objects used some are found to be more salient). Also in both experiments 1 and 2, D_1_ receptor stimulation decreases object exploration (as measured during the sampling phase). It could be argued that the NOR memory deficits observed relate to this reduction in exploratory encoding of the object to be remembered. In experiment 1 however, only the lowest dose of SKF81297 impaired memory, whereas (during the sampling phase) object exploration was decreased at both doses. If reduced sample exploration caused the apparent NOR impairment, both doses should have decreased NOR. In experiment 3, SKF81297 also decreased exploration of both the familiar and the novel object when injected before the test phase, possibly reflecting some locomotor effects. However the difference between the exploration times of the novel and familiar objects so clearly present in the saline group was absent after injection of SKF81297. Moreover, the pattern of results seen in experiment 1 shows that deficits in NOR are dissociable from non-specific locomotor effects.

### 4.2 Effects of DA D_1_ receptor stimulation on the encoding of NOR

Although DA has been implicated in recognition memory processing, its role in the different stages of this process – (encoding versus retrieval/expression) remain poorly understood. The results of experiments 1 and 2 show that SKF81297 impaired NOR when injected prior to the sampling phase and after a delay of 10 min and 24 h. At a short delay, it is impossible to discriminate whether memory formation or retrieval has been affected, the drug being effectively active in the brain during both processes. However, the persistence of the impairment after a delay of 24 h implies that the formation of a familiar memory is dependent on D_1_ receptor activation. Importantly, consolidation, which refers to a category of processes that stabilise a memory trace after its initial acquisition, has been demonstrated to be dependent on D_1_ receptor activation (de Lima et al., 2011). It is therefore possible that in experiments 1 and 2 the drug may have interfered with consolidation. That D_1_ receptor transmission may play a key role in NOR is in line with previous studies suggesting the importance of DA D_1_ receptor activation in different forms of memory (El-Ghundi et al., 2007) and memory encoding in particular (O׳Carroll et al., 2006, Pezze and Bast, 2012). Interestingly, NOR has been shown to rely on plasticity (Bussey et al., 1999, Brown and Aggleton, 2001) and the positive modulation by D_1_ receptor induction and maintenance of long-term potentiation (LTP) is well characterised (Jay, 2003, Huang et al., 2004, El-Ghundi et al., 2007). It has also been shown that a too much stimulation of the D_1_ receptor by SKF81297 increases signalling mediated by mTOR, a protein kinase involved in long lasting synaptic plasticity (Gangarossa et al., 2014;). Too much mTOR stimulation also blocks LTP and impairs long term recognition memory (Gangarossa et al., 2014). Together these findings suggest that the encoding of NOR memory may rely on the modulation of LTP by an optimal level of stimulation of the D_1_ receptor subtype. Our previous data showed that the D_3_ receptor antagonist S33084 alleviates the NOR deficit induced by social isolation (Watson et al., 2012a, Watson et al., 2012b). Our present results do not preclude the possibility that an appropriate dose of the D_1_ receptor agonist SKF81297 may act as a cognitive enhancer (Floresco and Phillips, 2001, Pezze et al., 2007; see also below).

### 4.3 Effects of DA D_1_ receptor stimulation on the retrieval of NOR

Besheer et al. (1999) have shown that systemic injection of the DA receptor antagonist SCH23390 before the retention test impaired performance of rats in detecting a novel object, suggesting an effect on retrieval. Hotte et al. (2005) reported that D_1_ receptor stimulation decreased NOR retrieval after a delay of 15 min. The present study confirmed that a similar dose of SKF81297 decreased performance after a delay of 10 min. However, the above study also reported enhanced retrieval after a long delay (Hotte et al., 2005), whereas the present results showed impairment NOR after a delay of 24 h. This may be related to procedural differences. First, in the present study, the animals were habituated to the empty arena on one occasion for one hour, whereas in the study of Hotte and co-workers, the habituation took place over 5 days in blocks of 3 to 10 min. Second, in our experiment the rats had 5 min to sample the objects compared to 3 min in the study by Hotte et al. (2005). Third, strain differences may have contributed to the different findings (Sprague–Dawley vs. Wistar rats). Thus, while SKF81297 clearly impairs NOR retrieval at a short retention delay of 10 min, the effects at longer retention delay of several hours are less clear cut.

### 4.4 Effects of DA D_1_ receptor stimulation in the PL mPFC on NOR memory

Pre-sampling infusions of SKF81297 into the PL part of the mPFC impaired NOR memory after a delay of 10 min without affecting activity or exploration. This suggest an involvement of D_1_ receptor activation in the formation and or retrieval/expression of NOR. Clausen et al. (2011) also reported a decrease in recognition memory after mPFC micro-infusion of the DA D_1_ receptor antagonist SCH23390. At first glance, these two set of data appear to be at odds with reports that excitotoxic lesions of the mPFC (Hannesson et al., 2004) and catecholaminergic depletion in the PL mPFC (Nelson et al., 2011) do not impair the standard NOR test in rodents. The lack of effect of these lesions may have been due to their incompleteness. In addition, permanent lesions, in contrast to temporary pharmacological blockade by drug microinfusion, may elicit compensatory process that lead to recovery of function (Bast and Feldon, 2003). In any event, negative results with lesions do not exclude a neuromodulatory role (Cassaday et al., 2014).

Together with Clausen׳s data, our results demonstrate that PL D_1_ receptor function is involved in non-spatial recognition memory (Clausen et al., 2011). The finding that both the D_1_ receptor antagonist SCH23390 (Clausen et al., 2011) and the agonist SKF81297 impaired NOR memory when infused into the PL may reflect an inverted U-shaped function relating the formation or retrieval/expression of NOR memory to DA-receptor stimulation in the mPFC. Similarly, working memory, attention and conditioned fear retrieval have also been suggested to require an optimal level of prefrontal DA-receptor stimulation (Williams and Goldman-Rakic, 1995, Arnsten, 1998, Robbins, 2000, Pezze et al., 2003).

Floresco and Phillips (2001) reported that activation of D_1_ receptors in the mPFC disrupts memory retrieval when performance is strengthened by a short delay. Interestingly, it has also been shown that impaired NOR retrieval by systemic injection of SCH23390 is correlated with decreased phosphorylation of cAMP response element binding protein (CREB) within the PFC (Hotte et al., 2006). Similarly, in conditioning procedures, Pezze et al. (2003) showed that the retrieval/expression but not formation of a conditioned emotional response (CER) relies on DA in the mPFC. Furthermore, it is also known that D_1_ receptor activation influences CER retrieval only (Lauzon et al., 2009). In the present study, infusions of SKF81297 in the PL sub-region of the mPFC before sampling impaired performance when tested 10 min later. Thus the drug was effective during both the encoding as well as the retrieval/expression phase of NOR and there was no clear dissociation between the memory stages. However, taken together with the results of the studies discussed above, impaired memory retrieval may be the major determinant of D_1_ receptor-induced NOR deficits.

## 5 Conclusions

The results of the present study show that DA D_1_ receptor activation modulates not only retrieval/expression, but also encoding in the NOR procedure. In addition, the results demonstrate that D_1_ receptor transmission within the PL part of the mPFC is important for NOR memory. The effects of prefrontal D_1_ receptor stimulation revealed in the present study resemble those reported following prefrontal D_1_ receptor antagonism in previous studies. Therefore, we propose that NOR memory requires an optimal level of D_1_ receptor stimulation in the mPFC.

## Role of funding source

This research was supported by a Grant from the BBSRC (Ref. BB/K004980/1). The BBSRC had no further involvement in the study design, in the collection, analysis or interpretation of data; in the writing of the report; nor in the decision to submit the paper for publication.

## Contributors

M.A. Pezze, H.J. Marshall, H.J. Cassaday designed and conceived the study. M.A. Pezze, H.J. Marshall, H.J. collected data and conducted statistical analysis. M.A. Pezze wrote the first draft of the manuscript. H.J. Cassaday, K.C.F Fone supervised the work. M.A. Pezze, H.J. Cassaday and K.C.F Fone critically reviewed the manuscript. All the authors contributed to and have approved the final manuscript.

## Conflict of interest

The authors declare no competing financial interests.

## Figures and Tables

**Table 1:** Effect of systemic injection or mPFC microinfusion of SKF81297 on exploration time in seconds (mean+S.E.M.) of both objects, during the sample (a), and choice (b) phases of a NOR task.

**Injections**	**Delay**	**Sal**	**0.4 mg/kg**	**0.8 mg/kg**
Systemic before encoding	10 min	a		
70.1±7.3	36.2±4.8 ⁎⁎	24.8±5.2⁎⁎⁎
b		
43.6±3.3	33.5±5.0	24.0±3.1⁎
Systemic before encoding	24 h	a		
37.6±5.5	20.6±4.8⁎	18.6±3.4⁎
b		
30.6±2.2	26.5±3.4	26.7±2.5
Systemic before test session^1^	24 h	a		
26.6±7.7	23.2±1.7	29.5±5.1
b		
24.2±3.1	7.5±1.6⁎⁎⁎	7.2±1.4 ⁎⁎⁎

**Infusions**	**Delay**	**Sal**	**0.025 µg/side**	**0.05 µg/side**
In mPFC before encoding	10 min	a		
56.3±4.1	45.0±5.2	56.1±8.4
b		
40.4±4.3	25.4±3.9⁎	33.4±4.6

⁎ *p*<0.05;

⁎⁎ *p*<0.001;

⁎⁎⁎ *p*<0.0001:significant decreases in the time spent exploring the objects compared to saline (sal).

1 Note: where drug administration was systemic before test session the sample exploration times of the untreated rats are provided by drug condition-to-be, for comparison.
