# Supplementary material for: Dopamine D1 receptor stimulation modulates the formation and retrieval of novel object recognition memory: Role of the prelimbic cortex
Source: Eur Neuropsychopharmacol. 2015 Nov;25(11):2145–56. doi: 10.1016/j.euroneuro.2015.07.018 (PMC4661036; doi:10.1016/j.euroneuro.2015.07.018)
Supplement: Supplementary file 1 — Supplementary data [file mmc1.doc]

**Supplementary material:**

Implantation of guide cannulae into the mPFC

Rats (n=11) were anesthetised using isoflurane delivered in oxygen (induction: 4-5%; maintenance: 1-3%) before being secured in a stereotaxic frame. The skull was exposed and bregma and lambda were aligned horizontally. A bilateral infusion guide cannula (model C235GS-5-1.2 Plastic Ones, Bilaney, UK) consisting of a 5 mm plastic pedestal holding two 26 gauge metal tubes 1.2 mm apart and projecting 4.5 mm from the pedestal was implanted through small holes drilled in the skull. The tips of the guide cannula were aimed 0.5 mm above the injection site in the PL part of the prefrontal cortex, at the following coordinates: 3 mm anterior and 0.6 mm lateral from bregma, and 3.8 mm ventral from the skull surface. These coordinates were adapted from a previous study (Marquis et al., 2007) on the basis of pilot surgeries. The cannula was secured to the skull with dental acrylic around stainless steel screws. Double stylets (33 gauge; Plastic Ones, Bilaney, UK) were inserted into the guides (with no protrusion) which was closed with a dust cap. Following surgery, rats were allowed at least 5 days recovery before any testing commenced. During the recovery period, rats were checked daily and habituated to the manual restraint necessary for the drug microinjection.

Behavioral apparatus

*Novel object recognition*

All testing was conducted in a 38 x 40 cm opaque plastic rectangular arena with 54 cm high walls. A video editing software (Pinacle studio 13) recorded from an overhead camera was used to record behaviour for subsequent analysis. The stimuli consisted of duplicate copies of bottles and flasks made of glass, metal or plastic of varied shape, colour and size which were too heavy to be displaced by the animal. During the sampling phase, two similar objects were placed in opposite corners of the arena. The sets of 2 objects used during the sampling phase differed markedly and did not appear to share common features. The particular set of objects selected was counterbalanced across tests, as was their placement (left or right of arena). The familiar object at test was always an identical copy of the object seen at sampling. Time spent exploring each object was defined as directing the nose at the object at a distance of less than 1 cm and actively exploring it (i.e. sniffing and or interacting with the object). Object exploration was not scored if the animal was in contact with but not facing the object or if it sat on the object or used it as a prop to look around or above the object (Ennaceur & Delacour, 1988). The test box and objects were cleaned with an alcohol-based solution (20% v/v) before each trial to remove odor cues.

*Locomotor activity*

Locomotor activity was measured in a dimly lit (50-70 Lux) room in 12 clear Perspex chambers (39.5 cm long x 23.5cm wide x 24.5 cm deep) with metal grid lids. The chambers were surrounded by frames containing two levels of photobeams (Photobeam Activity System, San Diego Instruments, USA) as described previously (Jones et al, 2011). Two consecutive breaks of adjacent beams within the lower level of photobeams generated a locomotor count. To start a session, rats were placed into the centre of the chamber. Total locomotor counts were recorded for each consecutive 10 min epoch for 60 min.

Histology

Following the completion of the experiments, rats were anesthetized with a lethal dose of sodium pentobarbitone (1-1.5 ml Euthatal; sodium pentobarbitone, 200 mg/ml; Genus Express, UK) and perfused transcardially with 0.9% saline followed by 4% formaldehyde solution in saline. Brains were removed from the skull, post-fixed in 4% formaldehyde, and cut into 80 μm coronal sections on a vibratome. Sections containing prefrontal cortex were mounted on slides and stained with cresyl violet. Placements of the injector were determined using a light microscope and mapped onto coronal sections of a rat brain stereotaxic atlas (Paxinos and Watson, 1998).
